# Troponin T and Neurofilament Light Chain Levels as Complementary Biomarkers of Disease Accumulation and Aggressiveness in Amyotrophic Lateral Sclerosis

**DOI:** 10.1002/acn3.70499

**Published:** 2026-07-23

**Authors:** Julia Sellin, Sofia Waldorf, Janina von der Gablentz, Torsten Grehl, Huelya Nazlican, Thomas Meyer, Julian Grosskreutz, Patrick Weydt, Sarah Bernsen

**Affiliations:** ^1^ University of Luebeck, Precision Neurology of Neuromuscular and Motor Neuron Disease Luebeck Germany; ^2^ Department of Neuromuscular Diseases, Center for Neurology University Hospital Bonn Bonn Germany; ^3^ Department of Neurology Alfried Krupp Hospital Essen Germany; ^4^ Department of Neurology, Center for ALS and Other Motor Neuron Disorders Charite‐Universitaetsmedizin Berlin, Corporate Member of Freie Universitaet Berlin, Humboldt‐Universitaet Zu Berlin and Berlin Institute of Health Berlin Germany; ^5^ Ambulanzpartner Soziotechnologie APST GmbH Berlin Germany; ^6^ University of Luebeck, Excellence Cluster for Precision Medicine in Inflammation Luebeck Germany; ^7^ German Center for Neurodegenerative Diseases Bonn Germany

**Keywords:** amyotrophic lateral sclerosis, biomarker, D50, neurofilament light chain, troponin T

## Abstract

**Objective:**

Amyotrophic lateral sclerosis (ALS) is a clinically heterogeneous neurodegenerative disease requiring reliable biomarkers to improve patient stratification and trial design. While serum neurofilament light chain (sNfL) reflects neuroaxonal stress and disease aggressiveness, troponin T (TnT) may capture complementary aspects of neuromuscular involvement. We assessed the associations of TnT and sNfL with D50‐derived measures of disease aggressiveness (D50) and disease accumulation (rD50) in ALS.

**Methods:**

In this retrospective observation, TnT and sNfL levels from ALS patients in two independent German cohorts were analyzed using the D50 disease progression model; discovery cohort (Essen, *n* = 433) and an independent replication cohort (Bonn, *n* = 185).

**Results:**

TnT levels were strongly associated with rD50‐defined disease phases in the discovery cohort (*p* < 0.001). While not all subgroup‐specific associations were replicated, the overall relationship between TnT and disease accumulation was supported in the independent replication cohort. In contrast, sNfL showed no consistent relationship with rD50‐derived disease phases. sNfL concentrations demonstrated a significant inverse association with D50, supporting a relationship with disease aggressiveness across both cohorts (*p* < 0.001). Associations between TnT levels and D50‐defined disease aggressiveness were generally weaker and less consistent.

**Interpretation:**

TnT was associated with measures of disease accumulation (rD50), whereas sNfL was more closely associated with disease aggressiveness (D50). Our results suggest that TnT and sNfL capture different dimensions of disease status within the D50 framework. Further longitudinal studies are needed to determine whether combining these biomarkers improves disease stratification or prognostic assessment in clinical practice and therapeutic trials.

## 1 Introduction

Amyotrophic lateral sclerosis (ALS) is a fatal neurodegenerative disease marked by progressive degeneration of upper and lower motor neurons [1]. Despite ongoing research, effective treatments remain scarce. The clinical course of ALS is highly heterogeneous, with substantial interindividual variability in disease progression and survival [2]. Reliable biomarkers are therefore needed to improve disease characterization, patient stratification, and clinical trial design [3].

Functional decline in ALS is commonly assessed using the revised ALS Functional Rating Scale (ALSFRS‐R) [4]. Disease aggressiveness is often estimated using the disease progression rate (DPR), derived from ALSFRS‐R decline. However, both measures have limitations, including susceptibility to measurement variability and the assumption of linear disease progression despite evidence for more complex longitudinal trajectories [5, 6, 7, 8, 9].

The D50 model was developed to account for heterogeneous progression patterns by fitting individual longitudinal ALSFRS‐R trajectories using a sigmoidal function [10, 11]. This framework allows the characterization of disease aggressiveness (D50) and disease accumulation (relative D50; rD50) as distinct dimensions of disease progression and has been applied in several ALS biomarker studies.

Here, the D50 framework was applied as an established disease progression model to examine biomarker associations, rather than to assess its performance relative to other progression metrics.

To date, neurofilaments represent the most established fluid biomarkers in ALS [12, 13]. Elevated neurofilament levels in serum and cerebrospinal fluid (CSF) reflect neuroaxonal injury, rise early in the disease course, and are associated with disease aggressiveness and survival [14]. Previous studies applying the D50 framework to CSF neurofilament light chain (NfL) and phosphorylated neurofilament heavy chain (pNfH) demonstrated strong associations with disease aggressiveness independent of disease accumulation [15, 16].

Troponin T (TnT), a well‐established cardiac biomarker that has been repurposed in neuromuscular disorders including ALS, has emerged as another promising biomarker candidate. Elevated TnT levels are common in ALS and are thought to reflect regenerative and/or degenerative skeletal muscle processes. In combination with sNfL, TnT may also improve diagnostic accuracy [17, 18, 19]. Furthermore, TnT levels increase longitudinally in untreated ALS patients and have been associated with functional decline, respiratory impairment, and lower motor neuron involvement [20, 21]. These observations suggest that TnT may capture aspects of ALS pathology distinct from those reflected by neurofilaments.

Thus far, NfL has emerged as a marker of disease aggressiveness within the D50 framework but appears less informative for disease accumulation. Whether TnT maps onto the same or a distinct dimension of the D50 framework has not been established.

We therefore applied the D50 model to two independent ALS cohorts to examine whether TnT is primarily associated with disease aggressiveness, as reflected by D50, or with time‐normalized disease accumulation, as reflected by rD50. By comparing these associations with those of serum NfL (sNfL), we sought to determine whether the two biomarkers relate to different dimensions of disease progression within the D50 framework.

## 2 Materials and Methods

### 2.1 Study Design

We performed a retrospective analysis of two independent patient cohorts from specialized ALS centers in Germany, comprising a discovery cohort from Alfried Krupp Hospital Essen and a replication cohort from University Hospital Bonn. All patients fulfilled the revised El Escorial criteria for possible, probable, or definite ALS including progressive muscle atrophy and had at least two clinical assessments to enable reliable D50 modeling.

Patients in the discovery cohort were seen in the ALS clinic between January 10, 2023, and April 1, 2025. The independent replication cohort had visits between January 1, 2020, and December 1, 2023. Data were extracted in both cohorts from routine clinical records and included demographic information (age, sex), clinical variables (ALSFRS‐R, site and date of onset), and biomarker levels (TnT, sNfL) taken at each visit. In each cohort, analyses included data starting from the first available TnT value following the implementation of routine TnT measurements.

Patients in the Essen cohort had provided written informed consent within the APST registry and the NfL‐ALS substudy (Ethics approvals EA2/168/20 and EA1/219/15).

No additional informed consent or ethical approval was required from the institutional ethics review board in Bonn, for the underlying data of this analysis was fully pseudonymized routine clinical data (Ethics board approval letter 2025/069 W, Bonn).

### 2.2 Laboratory Markers

sNfL concentrations (Essen) were measured at the ALS Center Berlin by means of the SIMOA, using the NfL Advantage Kit (Quanterix Inc., USA).

sNfL levels (Bonn) were measured at the University Medical Center Ulm using a standardized ELISA protocol [22].

Serum high‐sensitivity TnT was measured using an electrochemiluminescence immunoassay (ECLIA) at the central laboratory of Alfried Krupp Hospital (discovery cohort) and at an accredited commercial laboratory (Labor Volkmann, Karlsruhe, Germany) for the replication cohort.

### 2.3 D50 Model

The sigmoidal fit of the D50 model can be described with two parameters: first, D50 as time in months since initial symptom onset to the time point of 50% motor function loss, as loss of 24 points in the 48‐point ALSFRS‐R score and second, dx as time constant of functional decline, as steepness of the curve. As described before, D50 and dx correlate directly in different cohorts [23, 24].

The use of the D50 model enables robust comparison across heterogeneous patient cohorts by quantifying disease aggressiveness independently of disease duration at the time of assessment. Unlike conventional cross‐sectional metrics, this approach accounts for individual differences in baseline status and temporal disease evolution, allowing patients at different clinical stages to be aligned on a common progression scale. Consequently, stratification the patients participating in this analysis into disease aggressiveness subgroups of high (D50 < 20 months), intermediate (20 ≤ D50 < 40 months), and low (D50 ≥ 40 months) facilitates meaningful comparisons between cohorts by reducing bias introduced by variability in disease onset and observation time.

By normalizing the real‐time D50 value the rD50 can be calculated, a dimensionless parameter that maps disease progression on an open‐ended scale. An rD50 value of 0 is the time of disease onset, while a value of 0.5 represents the time point of 50% motor function loss; that is the D50.

This parameter can be calculated for any given time point during the disease course, enabling time‐normalized staging independent of individual progression speed.

With the help of rD50, we can categorize three disease phases: Phase I is designated as the early semi‐stable phase and is characterized by rD50 values ranging from 0 to 0.25. Phase II, the early progressive stable phase, is characterized by rD50 values ranging from 0.25 to 0.5. Phase III/IV, the late progressive and late stable phase, is defined by rD50 ≥ 0.5.

The D50 model further provides two descriptors of local disease activity at a given time point: the calculated functional loss rate (cFL), reflecting the steepness of the sigmoidal curve and expressed as points lost per month, and the calculated functional state (cFS), representing the estimated ALSFRS‐R score at a specific point along the individual disease trajectory. Because D50 has been extensively used as a measure of disease aggressiveness in previous applications of the D50 framework, it was selected as the primary disease aggressiveness parameter for the present analyses.

### 2.4 Statistics

Statistical analyses and graphical visualizations were performed using RStudio (Version 2023.06.01; Posit Software, PBC, Boston, MA, USA) and the R programming language on macOS Sequoia 15.5. The distribution of continuous variables was assessed using the Shapiro–Wilk test. sNfL, and TnT values were log‐transformed where appropriate to improve normality prior to parametric analyses.

To evaluate associations between biomarkers and D50‐derived disease progression measures, separate linear regression models were fitted with log‐transformed biomarker concentrations [ln(sNfL) or ln(TnT)] as dependent variables and either ln(D50) or rD50 as independent variables. Regression coefficients, *p*‐values, and coefficients of determination (R^2^) were calculated for each model.

Differences in sNfL and TnT concentrations across D50‐defined disease aggressiveness groups and rD50‐defined disease phases were assessed using analysis of covariance (ANCOVA), adjusting for age at onset, sex, and site of onset. Post hoc pairwise comparisons were performed using Wilcoxon rank‐sum tests with Bonferroni correction for multiple testing.

Primary analyses were based on the first available biomarker measurements. As a sensitivity analysis, all analyses were repeated using the last available sNfL and TnT measurements available for each patient.

## 3 Results

### 3.1 Patient Characteristics

Demographic, clinical data, DPR, ALSFRS‐R, and biomarker levels of the two cohorts are shown in Table 1 (Essen; discovery cohort) and Table 2 (Bonn; replication cohort) stratified by D50‐derived subgroups according to disease accumulation (rD50) and disease aggressiveness (D50).

**TABLE 1:** Demographic and clinical characteristics of the discovery cohort (Essen, *n* = 433).

Variable	Total cohort—essen		D50 disease aggressiveness			
	*N*	*N* = 433^a^	High *N* = 77^a^	Intermediate *N* = 148^a^	Low *N* = 208^a^	*p* ^b^
Sex						
Female	433	176 (41%)	38 (49%)	63 (43%)	75 (36%)	0.11
Age at Onset	428	63 (55, 70)	64 (57, 71)	66 (56, 73)	60 (54, 68)	< 0.001
Symptom onset Region						
Bulbar	433	70 (16%)	14 (18%)	34 (23%)	22 (11%)	0.002
Spinal		329 (76%)	53 (69%)	101 (68%)	175 (84%)	
Unknown		34 (7.9%)	10 (13%)	13 (8.8%)	11 (5.3%)	
First ALSFRS‐R Time since onset [months]	431	25 (13, 50)	10 (7, 14)	19 (13, 29)	47 (29, 97)	< 0.001
First ALSFRS‐R total score	431	35 (28, 41)	32 (27, 38)	35 (27, 40)	36 (29, 43)	0.004
Disease Progression Rate first ALSFRS‐R	433	0.45 (0.21, 0.85)	1.43 (1.10, 2.00)	0.63 (0.47, 0.82)	0.21 (0.12, 0.36)	< 0.001
cFS first ALSFRS‐R	431	36 (28, 41)	33 (29, 39)	36 (28, 41)	36 (28, 43)	0.052
cFL first ALSFRS‐R	430	0.60 (0.23, 0.98)	1.61 (1.33, 1.97)	0.82 (0.66, 0.96)	0.23 (0.12, 0.43)	< 0.001
D50 [months]	433	39 (24, 76)	15 (12, 18)	28 (24, 34)	81 (54, 174)	< 0.001
rD50 at first ALSFRS‐R	431	0.33 (0.22, 0.47)	0.37 (0.25, 0.44)	0.33 (0.23, 0.47)	0.32 (0.18, 0.47)	0.2
Disease phase first ALSFRS‐R						
I	431	139 (32%)	19 (25%)	45 (30%)	75 (36%)	0.4
II		203 (47%)	40 (52%)	72 (49%)	91 (44%)	
III/IV		89 (21%)	18 (23%)	31 (21%)	40 (19%)	
sNfL, first measure [pg/ml]	431	49 (27, 90)	103 (68, 167)	66 (42, 91)	30 (17, 49)	< 0.001
rD50 first sNfL measure	431	0.33 (0.22, 0.47)	0.37 (0.25, 0.50)	0.33 (0.23, 0.47)	0.32 (0.17, 0.47)	0.2
cFL first sNfL measure	430	0.60 (0.24, 0.98)	1.61 (1.31, 1.97)	0.82 (0.66, 0.97)	0.24 (0.12, 0.43)	< 0.001
cFS first sNfL measure	431	36 (28, 41)	33 (26, 39)	36 (28, 41)	36 (28, 43)	0.059
TnT, first measure [ng/L]	431	20 (11, 34)	21 (11, 36)	22 (13, 38)	17 (9, 31)	0.023
rD50 first TnT measure	431	0.33 (0.22, 0.47)	0.37 (0.25, 0.50)	0.33 (0.23, 0.47)	0.32 (0.18, 0.47)	0.2
cFL first TnT measure	430	0.60 (0.23, 0.98)	1.61 (1.33, 1.97)	0.82 (0.66, 0.96)	0.23 (0.12, 0.43)	< 0.001
cFS first TnT measure	431	36 (28, 41)	33 (26, 39)	36 (28, 41)	36 (28, 43)	0.046

*Note:* rD50, cFL and cFS first and last measure for ALSFRS‐R, sNFL and TnT.

Abbreviations: ALSFRS‐*R*, Amyotrophic Lateral Sclerosis Rating Scale‐Revised; cFL, calculated Functional Loss rate; cFS, calculated Functional State; n/*N*, Number; sNfL, serum Neurofilament light chain; TnT, Troponin T.

^a^ *n* (%); Median (Q1, Q3).

^b^ Pearson's Chi‐squared test, Kruskal‐Wallis rank sum test.

**TABLE 2:** Demographic and clinical characteristics of the replication cohort (Bonn).

Variable	Total cohort‐bonn		Splitted into D50 disease aggressiveness			
	*N*	*N* = 185^a^	high *N* = 48^a^	intermediate *N* = 63^a^	low *N* = 74^a^	*p* ^b^
Sex						
female	185	86 (46%)	22 (46%)	30 (48%)	34 (46%)	> 0.9
Age at Onset, years	185	60 (53, 69)	64 (55, 73)	62 (56, 70)	58 (51, 66)	0.018
Symptom Onset Region	185					0.050
bulbar		42 (23%)	15 (31%)	18 (29%)	9 (12%)	
spinal		133 (72%)	31 (65%)	43 (68%)	59 (80%)	
n.a.		10 (5.4%)	2 (4.2%)	2 (3.2%)	6 (8.1%)	
First ALSFRSR Time since onset [months]	185	18 (10, 35)	7 (5, 11)	17 (11, 20)	45 (25, 68)	< 0.001
First ALSFRS‐R total Score	185	37 (31, 41)	36 (29, 41)	38 (31, 40)	37 (31, 41)	0.5
Disease Progression Rate first ALSFRS‐R	185	0.57 (0.29, 1.05)	1.62 (1.11, 2.54)	0.67 (0.52, 0.92)	0.28 (0.18, 0.39)	< 0.001
D50 [months]	185	34 (20, 62)	14 (12, 17)	30 (25, 35)	64 (54, 104)	< 0.001
rD50 at first ALSFRS‐R	185	0.29 (0.20, 0.39)	0.26 (0.18, 0.36)	0.27 (0.20, 0.38)	0.32 (0.21, 0.40)	0.4
Disease phase first ALSFRS‐R	183					0.7
I		70 (38%)	20 (43%)	25 (40%)	25 (34%)	
II		96 (52%)	23 (49%)	34 (54%)	39 (53%)	
III/IV		17 (9.3%)	4 (8.5%)	4 (6.3%)	9 (12%)	
sNfL, first measure [pg/ml]	185	83 (52, 129)	124 (88, 202)	100 (63, 137)	53 (32, 77)	< 0.001
rD50, first sNfL measure	185	0.29 (0.20, 0.39)	0.27 (0.18, 0.36)	0.27 (0.20, 0.38)	0.32 (0.21, 0.40)	0.5
cFL_sNfL_first measure	185	0.65 (0.38, 1.12)	1.55 (1.28, 1.91)	0.80 (0.61, 1.03)	0.34 (0.24, 0.48)	< 0.001
cFS_sNfL_first measure	185	38 (32, 41)	38 (33, 41)	38 (33, 42)	37 (32, 42)	0.9
TnT, first measure [ng/L]	176	17 (9, 29)	18 (10, 34)	17 (8, 25)	18 (9, 32)	0.8
rD50 first TnT measure	185	0.29 (0.20, 0.39)	0.27 (0.18, 0.36)	0.29 (0.20, 0.38)	0.32 (0.21, 0.40)	0.5
cFL_TnT_first measure	185	0.65 (0.38, 1.12)	1.55 (1.27, 1.91)	0.80 (0.61, 1.04)	0.34 (0.23, 0.48)	< 0.001
cFS_TnT_first measure	185	38 (32, 41)	38 (33, 41)	39 (33, 42)	37 (32, 42)	0.9

*Note:* rD50, cFL and cFS first measure for ALSFRS‐R, sNFL and TnT.

Abbreviations: ALSFRS‐*R*, Amyotrophic Lateral Sclerosis Rating Scale‐Revised; cFL, calculated Functional Loss rate; cFS, calculated Functional State; n/*N*, Number; sNfL, serum Neurofilament light chain; TnT, Troponin T.

^a^ *n* (%); Median (Q1, Q3).

^b^ Pearson's Chi‐squared test, Kruskal‐Wallis rank sum test, Fisher's exact test.

Sex distribution was similar between the discovery (*n* = 433) and replication cohort (*n* = 185) (*p* = 0.210). Patients in the discovery cohort were older with a median age at onset of 63 years (interquartile range (IQR) 55–70) vs. 60 years (IQR 53–69) (*p* = 0.052).

The discovery cohort exhibited greater functional impairment, reflected by lower median ALSFRS‐R scores (*p* = 0.020) and cFS (*p* = 0.003), compared with the replication cohort. At the same time, disease progression measures differed between cohorts, with lower DPR (*p* = 0.005), lower cFL (*p* = 0.002), and higher D50 values (39 months [IQR 24–76] vs. 34 months [IQR 20–62]; *p* = 0.009) observed in the discovery cohort. These findings indicate differences in disease severity and disease aggressiveness distributions between the two cohorts. A higher proportion of patients was classified into the low‐aggressiveness subgroup in the discovery cohort than in the replication cohort (48% vs. 40%). Consistent with this distribution, median rD50 at first sampling was higher in the discovery cohort than in the replication cohort (0.330 [IQR 0.22–0.47] vs. 0.290 [IQR 0.20–0.39]; *p* < 0.001), resulting in differences in the distribution of rD50‐defined disease phases. Accordingly, a greater proportion of patients in the discovery cohort presented in advanced disease phases (Phase III/IV) compared with the replication cohort (21% vs. 9.3%).

Consistent with previous reports, patients with lower disease aggressiveness (higher D50 values) tended to be sampled at earlier stages of disease accumulation than patients with higher disease aggressiveness (lower D50 values), reflecting the well‐described sampling shift inherent to the D50 framework [15, 25].

### 3.2 Biomarkers and Disease Aggressiveness (D50)

Across both cohorts, ANCOVA identified disease aggressiveness as the principal factor associated with sNfL concentrations and disease accumulation as the principal factor associated with TnT concentrations (both *p* < 0.001). Additional covariates, including age and site of onset for sNfL and sex for TnT, demonstrated less consistent effects and generally lower F statistics. Results of the adjusted ANCOVA models are provided in Table S1.

#### 3.2.1 sNfL and D50

**FIGURE 1:**
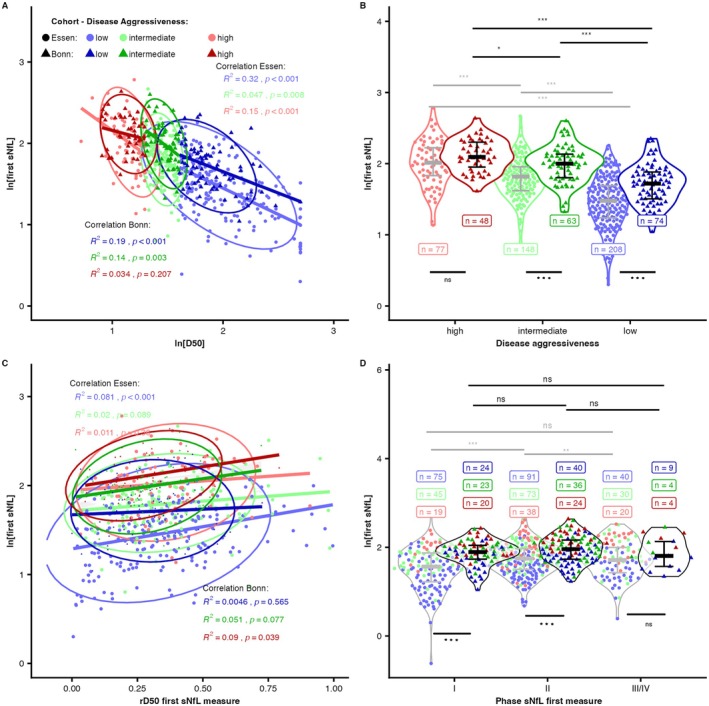
Associations of serum neurofilament light chain (sNfL) with disease aggressiveness and disease accumulation in the discovery cohort (Essen, *n* = 433) and independent replication cohort (Bonn, *n* = 185). (A) Correlation of first ln[sNFL] and ln[D50] with *p*‐values and R^2^ values as well as circles as 95% confidence interval and lines for linear regression for disease aggressiveness. (B) First measured ln[sNFL] split into disease aggressiveness and indication of significance levels within each cohort. (C) Correlation between ln[sNFL] concentrations at first measurement and relative D50 (rD50) as well as circles as 95% confidence interval and lines for linear regression for rD50. (D) ln[sNFL] across rD50‐defined disease phases (Phase I, Phase II, and Phase III/IV). Light colors and circles indicate the discovery cohort; dark colors and triangles indicate the independent replication cohort. Disease aggressiveness groups were defined as high (D50 < 20 months), intermediate (D50 20–40 months), and low (D50 ≥ 40 months). Statistical analyses are described in the Methods section.

In the discovery cohort, ln(sNfL) concentrations showed a significant inverse association with D50 across low‐ and high disease aggressiveness subgroups (*p* < 0.001) and intermediate subgroup (*p* = 0.008). In the replication cohort, this association remained significant in the intermediate (*p* < 0.001) and low‐aggressiveness subgroups (*p* = 0.003; Figure 1A).

Comparison of sNfL concentrations across D50‐defined disease aggressiveness groups revealed significant differences between all groups in the discovery cohort (all *p* < 0.005). In the replication cohort, sNfL concentrations also differed significantly between disease aggressiveness groups (*p* < 0.005), although the distinction between the high‐ and intermediate‐aggressiveness groups was less pronounced (*p* < 0.05; Figure 1B).

#### 3.2.2 TnT and D50

**FIGURE 2:**
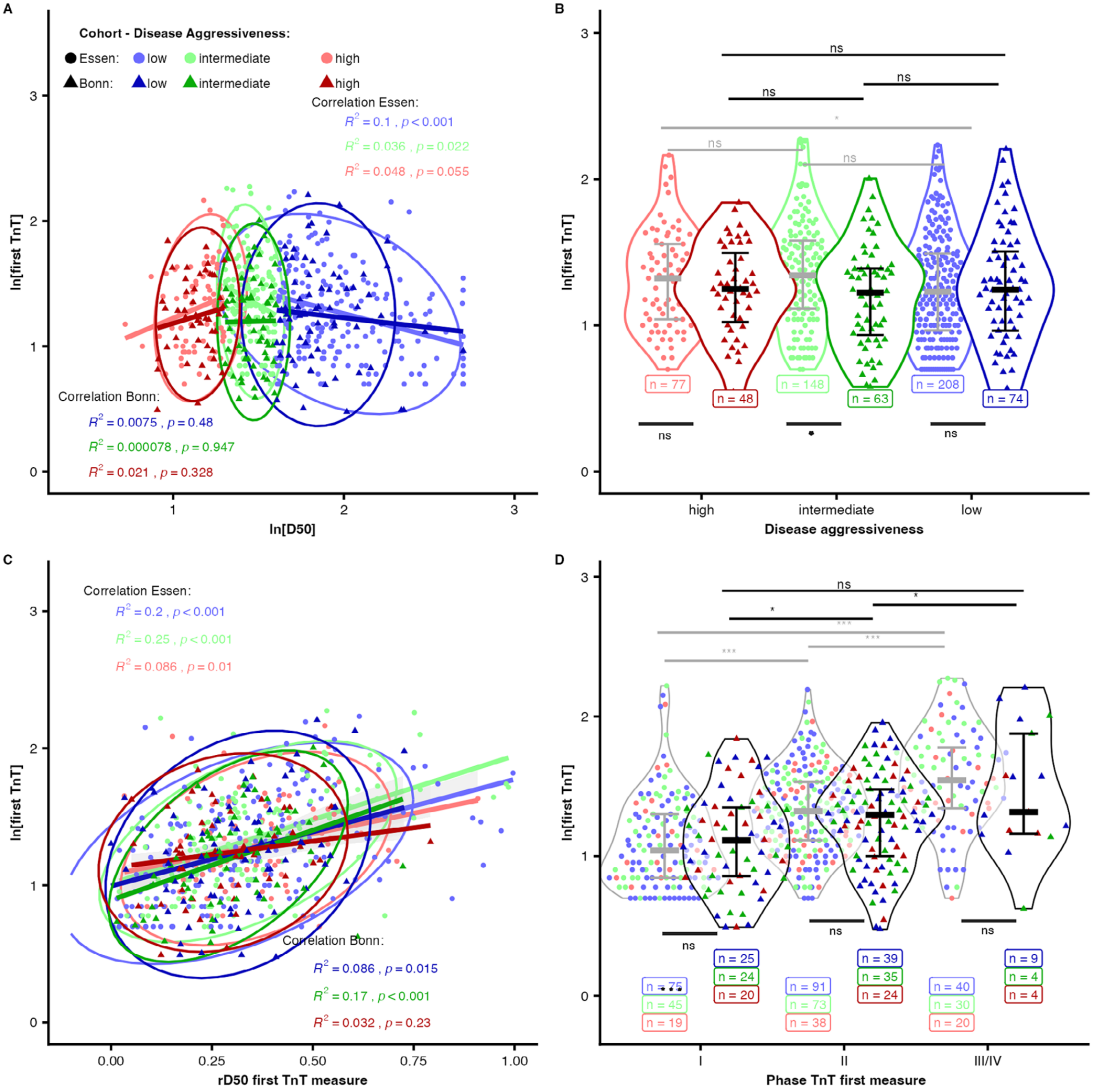
Associations of serum troponin T (TnT) with disease aggressiveness and disease accumulation in the discovery cohort (Essen, *n* = 433) and independent replication cohort (Bonn, *n* = 185). (A) Correlation of first ln[TnT] and ln[D50] with *p*‐values and R^2^—values as well as circles as 95% confidence interval and lines for linear regression for disease aggressiveness. (B) First measured ln[TnT] split into disease aggressiveness and indication of significance levels within each cohort. (C) Correlation between ln[TnT] concentrations at first measurement and relative D50 (rD50) as well as circles as 95% confidence interval and lines for linear regression for rD50. (D) ln[TnT] across rD50‐defined disease phases (Phase I, Phase II, and Phase III/IV). Light colors and circles indicate the discovery cohort; dark colors and triangles indicate the independent replication cohort. Disease aggressiveness groups were defined as high (D50 < 20 months), intermediate (D50 20–40 months), and low (D50 ≥ 40 months). Statistical analyses are described in the Methods section.

Associations between ln(TnT) concentrations and D50‐defined disease aggressiveness were generally weaker and less consistent across cohorts than those observed for sNfL. In the discovery cohort, a significant inverse association was observed in the low‐aggressiveness subgroup (*p* < 0.001), whereas only a weak association was present in the intermediate‐aggressiveness subgroup (*p* = 0.022). No significant association was detected in the high‐aggressiveness subgroup. In the replication cohort, ln(TnT) concentrations were not significantly associated with D50 in any disease aggressiveness subgroup (Figure 2A).

Consistent with these findings, TnT concentrations did not differ significantly across D50‐defined disease aggressiveness groups in the discovery cohort, with the exception of a difference between the intermediate‐ and low‐aggressiveness groups (*p* = 0.020). No significant differences were observed between disease aggressiveness groups in the replication cohort (Figure 2B).

For comparison with a conventional progression metric, exploratory analyses using DPR were performed. sNfL concentrations correlated significantly with DPR in both the discovery and replication cohorts (both *p* < 0.001). TnT concentrations correlated significantly with DPR only in the discovery cohort (*p* < 0.001), whereas no significant association was observed in the replication cohort (*p* = 0.541).

### 3.3 Biomarkers and Disease Accumulation (rD50)

#### 3.3.1 sNfL and rD50

No consistent association between ln(sNfL) concentrations and rD50‐defined disease phases was observed across cohorts. In the discovery cohort, ln(sNfL) concentrations were not significantly associated with rD50 in the intermediate‐ or high‐aggressiveness subgroups (both *p* > 0.05), whereas a significant association was observed in the low‐aggressiveness subgroup (*p* < 0.001; Figure 1C).

When comparing rD50‐defined disease phases, sNfL concentrations differed significantly between Phase I and Phase II (*p* < 0.001) and between Phase II and Phase III/IV (*p* < 0.001), but not between Phase I and Phase III/IV in the discovery cohort (Figure 1D).

In the replication cohort, ln(sNfL) concentrations showed only a weak association with rD50 in the high‐aggressiveness subgroup (*p* = 0.039), whereas no significant associations were observed in the intermediate‐ or low‐aggressiveness subgroups (Figure 1C). There was no significant difference between disease phases (Figure 1D).

#### 3.3.2 TnT and rD50

In the discovery cohort, ln(TnT) concentrations were significantly associated with rD50 across all disease aggressiveness subgroups (all *p* < 0.001; Figure 2C). Consistent with these findings, TnT concentrations differed significantly between all rD50‐defined disease phases in the discovery cohort (all *p* < 0.001; Figure 2D).

In the replication cohort, the association between ln(TnT) concentrations and rD50 was confirmed in the intermediate‐ (*p* < 0.001) and low‐aggressiveness subgroups (*p* = 0.015) but not in the high‐aggressiveness subgroup (*p* = 0.230; Figure 2C). Likewise, TnT concentrations differed significantly between Phase I and Phase II (*p* = 0.010) and between Phase II and Phase III/IV (*p* = 0.010), whereas no significant difference was observed between Phase I and Phase III/IV (Figure 2D).

To assess whether the observed associations were influenced by the choice of sampling time point, all analyses were repeated using the last available sNfL and TnT measurements in both cohorts. The overall patterns were comparable to those observed for the first available biomarker measurements, indicating that the findings were not dependent on the selected sampling time point. Detailed results are (shown in Figure S1 discovery cohort and Figure S2 replication cohort).

## 4 Discussion

Our study examined the relationships of serum TnT and sNfL with disease aggressiveness and disease accumulation within the D50 framework in two independent ALS cohorts. The principal finding is that TnT and sNfL showed distinct association patterns with D50‐derived measures of disease progression. Whereas sNfL was primarily associated with disease aggressiveness (D50), TnT showed more consistent associations with disease accumulation (rD50), suggesting that these biomarkers may capture complementary aspects of ALS disease biology.

The observed association between TnT and disease accumulation is biologically plausible. Previous studies have shown that TnT levels increase during the course of ALS and are associated with functional decline and lower motor neuron involvement [20, 21, 26, 27]. Within the D50 framework, these findings are consistent with an association between TnT and cumulative disease‐related changes within the motor unit. Similar observations have been reported for lower motor neuron–related measures such as MUNIX [28, 29].

The association between TnT and rD50 was observed across all disease aggressiveness subgroups in the discovery cohort. However, replication in the independent cohort was incomplete, as associations were confirmed in the intermediate‐ and low‐aggressiveness subgroups but not in the high‐aggressiveness subgroup.

Associations between TnT and disease aggressiveness (D50) are weaker and less consistent than those observed for disease accumulation and showed limited reproducibility across cohorts. Significant associations were largely confined to patients with low disease aggressiveness in the discovery cohort and were not confirmed in the independent cohort. These differences may reflect cohort‐specific characteristics, as the low aggressiveness subgroup in the discovery cohort demonstrates a less aggressive disease course, characterized by both lower DPR and cFL as well as higher D50 values. Similarly, Lindenborn et al. [19] reported that patients with ALS exhibiting concurrently low TnT and sNfL levels represent a distinct subgroup with a minimally aggressive phenotype.

Sex was independently associated with TnT concentrations in both cohorts, consistent with known sex‐related differences in troponin levels and other muscle‐related biomarkers [30, 31]. Adjustment for sex did not alter the association between TnT and rD50, suggesting that sex contributes to variability in TnT concentrations but does not fully explain the observed relationship. Future studies should evaluate sex‐specific TnT distributions and determine whether sex‐specific reference ranges or cutoffs improve biomarker interpretation in ALS.

The interpretation of TnT as a marker of disease accumulation is supported by previous longitudinal studies demonstrating increasing TnT concentrations over the disease course, associations with functional decline and respiratory impairment, and stabilization under disease‐modifying therapy. Although the present study was not designed to assess longitudinal biomarker dynamics, similar findings were observed using both the first and last available biomarker measurements. Notably, this represents an additional cross‐sectional analysis rather than a longitudinal evaluation of biomarker trajectories and was performed solely to assess the stability of the observed associations across different sampling time points. Future prospective studies are needed to determine whether TnT can reliably monitor disease accumulation and treatment response over time.

As detailed cardiometabolic comorbidity data were not uniformly available across both retrospective cohorts, residual confounding by non‐ALS determinants of TnT could not be fully excluded and is addressed as a limitation.

By contrast, sNfL was closely linked to disease aggressiveness in both cohorts, extending previous observations from CSF neurofilament studies within the D50 framework [15, 16]. This finding is in line with the established role of neurofilaments as markers of neuroaxonal injury and disease aggressiveness in ALS [12, 13, 14, 15, 16]. Relationships between sNfL and rD50‐defined disease phases were less pronounced. While some analyses identified differences between early and later disease phases in the discovery cohort, these findings were not replicated in the independent cohort. A possible explanation is that the discovery cohort is much larger and has substantially more patients in phase I with a low aggressive disease course than in phases II and III/IV. More broadly, the composition of aggressiveness groups differed substantially between cohorts, indicating that application of the D50 model reveals significant inter‐center differences in patient enrolment and cohort structure. Notably, sNfL concentrations in the replication cohort were significantly higher in the intermediate and low aggressiveness subgroups, as well as in phases I and II. This finding may reflect differences in assay platforms, disease aggressiveness distribution, and bulbar onset frequency between cohorts [14].

Taken together, the results support the concept that neurofilament levels primarily reflect disease aggressiveness rather than cumulative disease burden.

Several factors should be considered when interpreting comparisons between cohorts. Differences in disease aggressiveness, disease phase distribution, and biomarker assessment procedures may have contributed to inter‐cohort variability. In particular, the use of different sNfL assay platforms (SIMOA vs. ELISA) limits direct comparisons of absolute concentrations. TnT measurements were performed in different laboratories but using standardized high‐sensitivity clinical assays, making substantial inter‐laboratory variability less likely. Accordingly, the two cohorts were analyzed primarily as separate discovery and replication datasets, and the principal conclusions are based on the consistency of within‐cohort association patterns rather than on direct comparisons of absolute biomarker levels. Notably, the overall association patterns were similar across both cohorts despite substantial methodological and clinical differences.

The retrospective design restricted the availability of detailed clinical variables, including comprehensive measures of upper and lower motor neuron burden, cognitive involvement, respiratory function, and genetic status. Furthermore, survival analyses were not feasible because comprehensive longitudinal survival data were not uniformly available across both real‐world cohorts. Future prospective studies will be required to determine whether TnT provides incremental prognostic value.

Our findings do not establish a novel biological role for either biomarker and they were not intended to compare the D50 framework with alternative measures of disease progression, such as DPR or survival outcomes. Rather, our data suggest that previously described biomarker characteristics can be mapped onto different dimensions of disease progression captured by the D50 framework, an established disease‐course model. Additional exploratory analyses using DPR did not alter the principal interpretation of the study, as sNfL showed consistent associations with both D50‐derived measures and DPR, whereas associations between TnT and DPR were less reproducible across cohorts. These findings suggest that D50‐derived measures and conventional progression metrics may capture partially distinct aspects of disease progression. Whether this distinction provides clinically meaningful information beyond conventional progression metrics remains to be determined.

An important aspect is that conventional clinical measures frequently conflate disease aggressiveness and disease stage. Patients with comparable ALSFRS‐R scores may differ substantially in the rate at which disability accumulated, whereas patients with similar progression rates may present at markedly different stages of disease.

An additional conceptual limitation relates to the dependence of D50‐derived measures on longitudinal ALSFRS‐R trajectories. Because D50 and rD50 are calculated from repeated ALSFRS‐R assessments, associations between biomarkers and these parameters cannot be considered entirely independent of functional impairment measures. Consequently, the present findings should be interpreted within the context of the D50 framework rather than as evidence of completely independent biological processes. However, the distinct association patterns observed for sNfL and TnT argue against a purely methodological explanation. Nevertheless, further validation across independent clinical and biological outcomes will be important to establish their significance.

## 5 Conclusion

By applying the D50 framework, the present study demonstrates that two ALS biomarkers relate differently to distinct dimensions of disease progression. These findings suggest that sNfL and TnT may provide complementary information when interpreted within a disease‐course model that separates disease aggressiveness from disease stage.

Despite differences between cohorts, comparable association patterns were observed across independent datasets.

Future prospective studies should determine whether this information improves disease monitoring, prognostic assessment, and patient stratification beyond established clinical and biomarker measures.

## Author Contributions

Patrick Weydt, Julian Grosskreutz, Janina von der Gablentz, Sofia Waldorf, and Sarah Bernsen conceived of and designed the study. Torsten Grehl, Huelya Nazlican, Patrick Weydt, Sarah Bernsen, Julian Grosskreutz, Julia Sellin, Janina von der Gablentz, and Thomas Meyer contributed to acquisition and analysis of the data. Sofia Waldorf, Sarah Bernsen, Julian Grosskreutz, Patrick Weydt, and Julia Sellin drafted the text and prepared the figures.

## Funding

The authors have nothing to report.

## Conflicts of Interest

The authors declare no conflicts of interest.

## Supporting information


**Figure S1:** Discovery Cohort (Essen, light Colors and circles, *n* = 433) and Replication Cohort (Bonn, dark colors and triangles, *n* = 185) with serum NfL (sNfL) at last measure. (A) Correlation of last ln[sNFL] and ln[D50] with *p*‐values and R^2^—values as well as circles as 95% Confidence‐interval and lines for linear regression for disease aggressiveness. (B) Last measured ln[sNFL] splitted into disease aggressiveness and indication of significance levels within each cohort (C) Correlation between ln[sNFL] concentrations at last measurement and relative D50 (rD50) as well as circles as 95% confidence‐interval and lines for linear regression for rD50. (D) ln[sNFL] across rD50‐defined disease phases (Phase I, Phase II, and Phase III/IV).
Light colors and circles indicate the discovery cohort; dark colors and triangles indicate the independent replication cohort. Disease aggressiveness groups were defined as high (D50 < 20 months), intermediate (D50 20–40 months), and low (D50 ≥ 40 months). Statistical analyses are described in the Methods section.

**Figure S2:** Discovery Cohort (Essen, light Colors and circles, *n* = 433) and Replication Cohort (Bonn, dark Colors and triangles, *n* = 185) Troponin T (TnT) at last measure. (A) Correlation of last ln[TnT] and ln[D50] with *p*‐values and R^2^—values as well as circles as 95% Confidence‐interval and lines for linear regression for disease aggressiveness. (B) Last measured ln[TnT] splitted into disease aggressiveness and indication of significance levels within each cohort (C) Correlation between ln[TnT] concentrations at last measurement and relative D50 (rD50) as well as circles as 95% confidence‐interval and lines for linear regression for rD50. (D) ln[TnT] across rD50‐defined disease phases (Phase I, Phase II, and Phase III/IV).
Light colors and circles indicate the discovery cohort; dark colors and triangles indicate the independent replication cohort. Disease aggressiveness groups were defined as high (D50 < 20 months), intermediate (D50 20–40 months), and low (D50 ≥ 40 months). Statistical analyses are described in the Methods section.

**Table S1:** ANCOVA with first sNFL and first TnT with Cofactors: Age at Onset, rD50, D50, Sex and Symptom Onset Region with effect size (Eta square and omega).

## Data Availability

All data will be made available upon reasonable request to the corresponding author, SB.

## References

[1] E. L. Feldman , S. A. Goutman , S. Petri , et al., “Amyotrophic Lateral Sclerosis,” Lancet 400 (2022): 1363–1380, 10.1016/S0140-6736(22)01272-7. 36116464 PMC10089700

[2] A. Chiò , A. Calvo , C. Moglia , L. Mazzini , and G. Mora , “Phenotypic Heterogeneity of Amyotrophic Lateral Sclerosis: A Population Based Study,” Journal of Neurology, Neurosurgery, and Psychiatry 82 (2011): 740–746, 10.1136/jnnp.2010.235952. 21402743

[3] R. Chia , R. Moaddel , J. Y. Kwan , et al., “A Plasma Proteomics‐Based Candidate Biomarker Panel Predictive of Amyotrophic Lateral Sclerosis,” Nature Medicine 31 (2025): 3440–3450, 10.1038/s41591-025-03890-6. PMC12532604 40830661

[4] J. M. Cedarbaum , N. Stambler , E. Malta , et al., “The ALSFRS‐R: A Revised ALS Functional Rating Scale That Incorporates Assessments of Respiratory Function. BDNF ALS Study Group (Phase III),” Journal of the Neurological Sciences 169 (1999): 13–21, 10.1016/s0022-510x(99)00210-5. 10540002

[5] S. Paganoni , M. Cudkowicz , and J. D. Berry , “Outcome Measures in Amyotrophic Lateral Sclerosis Clinical Trials,” Clin Investig (Lond) 4 (2014): 605–618, 10.4155/cli.14.52. PMC5305182 28203356

[6] R. P. A. van Eijk , A. D. de Jongh , S. Nikolakopoulos , et al., “An Old Friend Who Has Overstayed Their Welcome: The ALSFRS‐R Total Score as Primary Endpoint for ALS Clinical Trials,” Amyotrophic Lateral Sclerosis and Frontotemporal Degeneration 22 (2021): 300–307, 10.1080/21678421.2021.1879865. 33527843

[7] R. P. A. van Eijk , D. N. Weemering , S. Opie‐Martin , et al., “Natural History of the Revised ALS Functional Rating Scale and Its Association With Survival: The PRECISION‐ALS Extant Study,” Amyotroph Lateral Scler Frontotemporal Degener 26 (2025): 30–40, 10.1080/21678421.2024.2443985. 40326917

[8] L. A. Bakker , C. D. Schröder , H. H. G. Tan , et al., “Development and Assessment of the Inter‐Rater and Intra‐Rater Reproducibility of a Self‐Administration Version of the ALSFRS‐R,” Journal of Neurology, Neurosurgery, and Psychiatry 91 (2020): 75–81, 10.1136/jnnp-2019-321138. 31558653

[9] M. Proudfoot , A. Jones , K. Talbot , A. al‐Chalabi , and M. R. Turner , “The ALSFRS as an Outcome Measure in Therapeutic Trials and Its Relationship to Symptom Onset,” Amyotroph Lateral Scler Frontotemporal Degener 17 (2016): 414–425, 10.3109/21678421.2016.1140786. 26864085 PMC4950444

[10] J. Meyer , N. Gaur , J. Gablentz , et al., “Phosphorylated Neurofilament Heavy Chain (pNfH) Concentration in Cerebrospinal Fluid Predicts Overall Disease Aggressiveness (D50) in Amyotrophic Lateral Sclerosis,” Frontiers in Neuroscience 19 (2025): 1536818, 10.3389/fnins.2025.1536818. 40143847 PMC11936903

[11] K. Poesen , M. Schaepdryver , B. Stubendorff , et al., “Neurofilament Markers for ALS Correlate With Extent of Upper and Lower Motor Neuron Disease,” Neurology 88 (2017): 2302–2309, 10.1212/WNL.0000000000004029. 28500227

[12] M. Benatar , E. A. Macklin , A. Malaspina , et al., “Prognostic Clinical and Biological Markers for Amyotrophic Lateral Sclerosis Disease Progression: Validation and Implications for Clinical Trial Design and Analysis,” eBioMedicine 108 (2024): 105323, 10.1016/j.ebiom.2024.105323. 39270623 PMC11415817

[13] K. Poesen and P. van Damme , “Diagnostic and Prognostic Performance of Neurofilaments in ALS,” Frontiers in Neurology 9 (2018): 1167, 10.3389/fneur.2018.01167. 30713520 PMC6345692

[14] F. Verde , P. Steinacker , J. H. Weishaupt , et al., “Neurofilament Light Chain in Serum for the Diagnosis of Amyotrophic Lateral Sclerosis,” Journal of Neurology, Neurosurgery, and Psychiatry 90, no. 2 (2019): 157–164, 10.1136/jnnp-2018-318704. 30309882

[15] M. Dreger , R. Steinbach , N. Gaur , et al., “Cerebrospinal Fluid Neurofilament Light Chain (NfL) Predicts Disease Aggressiveness in Amyotrophic Lateral Sclerosis: An Application of the D50 Disease Progression Model,” Frontiers in Neuroscience 15 (2021): 651651, 10.3389/fnins.2021.651651. 33889072 PMC8056017

[16] R. Steinbach , N. Gaur , A. Roediger , et al., “Disease Aggressiveness Signatures of Amyotrophic Lateral Sclerosis in White Matter Tracts Revealed by the D50 Disease Progression Model,” Human Brain Mapping 42 (2021): 737–752, 10.1002/hbm.25258. 33103324 PMC7814763

[17] M. Vidovic , H. S. Lapp , C. Weber , et al., “Comparative Analysis of Neurofilaments and Biomarkers of Muscular Damage in Amyotrophic Lateral Sclerosis,” Brain Commun 6 (2024): fcae288, 10.1093/braincomms/fcae288. 39239150 PMC11375854

[18] S. Castro‐Gomez , B. Radermacher , P. Tacik , S. R. Mirandola , M. T. Heneka , and P. Weydt , “Teaching an Old Dog New Tricks: Serum Troponin T as a Biomarker in Amyotrophic Lateral Sclerosis,” Brain Commun 3 (2021): fcab274, 10.1093/braincomms/fcab274. 34993474 PMC8728713

[19] P. Lindenborn , R. Fabian , T. Grehl , et al., “Combination of Serum Neurofilament Light Chain and Serum Cardiac Troponin T as Biomarkers Improves Diagnostic Accuracy in Amyotrophic Lateral Sclerosis,” Annals of Neurology 99, no. 2 (2026): 408–417, 10.1002/ana.78066. 41133969 PMC12894507

[20] T. Koch , R. Fabian , L. Weinhold , et al., “Cardiac Troponin T as a Serum Biomarker of Respiratory Impairment in Amyotrophic Lateral Sclerosis,” Annals of Clinical Translational Neurology 11 (2024): 2063–2072, 10.1002/acn3.52126. 38923228 PMC11330226

[21] S. Bernsen , R. Fabian , Y. Koc , et al., “Serum Cardiac Troponin T Levels as a Therapy Response Marker in Tofersen‐Treated ALS,” Muscle & Nerve 72 (2025): 509–514, 10.1002/mus.28453. 40491248 PMC12338008

[22] P. Steinacker , E. Feneberg , J. Weishaupt , et al., “Neurofilaments in the Diagnosis of Motoneuron Diseases: A Prospective Study on 455 Patients,” Journal of Neurology, Neurosurgery, and Psychiatry 87 (2016): 12–20, 10.1136/jnnp-2015-311387. 26296871

[23] N. Gaur , R. Steinbach , M. Plaas , O. W. Witte , M. S. Brill , and J. Grosskreutz , “Chitinase Dysregulation Predicts Disease Aggressiveness in ALS: Insights From the D50 Progression Model,” Journal of Neurology, Neurosurgery, and Psychiatry 94 (2023): 585–588, 10.1136/jnnp-2022-330318. 37076292

[24] R. Steinbach , M. Batyrbekova , N. Gaur , et al., “Applying the D50 Disease Progression Model to Gray and White Matter Pathology in Amyotrophic Lateral Sclerosis,” Neuroimage Clin 25 (2020): 102094, 10.1016/j.nicl.2019.102094. 31896467 PMC6940701

[25] M. Dreger , R. Steinbach , M. Otto , M. R. Turner , and J. Grosskreutz , “Cerebrospinal Fluid Biomarkers of Disease Activity and Progression in Amyotrophic Lateral Sclerosis,” Journal of Neurology, Neurosurgery, and Psychiatry 93, no. 4 (2022): 422–435. 35105727 10.1136/jnnp-2021-327503 PMC8921583

[26] U. Kläppe , S. Chamoun , Q. Shen , et al., “Cardiac Troponin T Is Elevated and Increases Longitudinally in ALS Patients,” Amyotroph Lateral Scler Frontotemporal Degener 23 (2022): 58–65, 10.1080/21678421.2021.1939384. 34151677

[27] S. Chamoun , S. Imrell , Z. Upate , et al., “Plasma Troponin T Reflects Lower Motor Neuron Involvement on Electromyography in Amyotrophic Lateral Sclerosis,” Brain Commun 7 (2025): fcaf177, 10.1093/braincomms/fcaf177. 40385376 PMC12082033

[28] T. Ebersbach , A. Roediger , R. Steinbach , et al., “Motor Unit Number Index (MUNIX) Loss of 50% Occurs in Half the Time of 50% Functional Loss According to the D50 Disease Progression Model of ALS,” Scientific Reports 13 (2023): 3981, 10.1038/s41598-023-30871-x. 36894607 PMC9998642

[29] T. Ebersbach , A. Roediger , R. Steinbach , et al., “Motor Unit Number Index (MUNIX) in the D50 Disease Progression Model Reflects Disease Accumulation Independently of Disease Aggressiveness in ALS,” Scientific Reports 12 (2022): 15997, 10.1038/s41598-022-19911-0. 36163485 PMC9512899

[30] D. M. Kimenai , A. S. V. Shah , D. A. McAllister , et al., “Sex Differences in Cardiac Troponin I and T and the Prediction of Cardiovascular Events in the General Population,” Clinical Chemistry 67, no. 10 (2021): 1351–1360, 10.1093/clinchem/hvab109. 34240125 PMC8486023

[31] J. C. Cook , E. Wong , and L. J. Haywood , “Creatine Kinase: Race‐Gender Differences in Patients Hospitalized for Suspected Myocardial Infarction,” Journal of the National Medical Association 82, no. 4 (1990): 249–254. 2185368 PMC2626127

