# Supplementary figures and images for: Troponin T and Neurofilament Light Chain Levels as Complementary Biomarkers of Disease Accumulation and Aggressiveness in Amyotrophic Lateral Sclerosis
Source: Ann Clin Transl Neurol. 2026 Jul 23:10.1002/acn3.70499. Online ahead of print. doi: 10.1002/acn3.70499 (PMC13396867; doi:10.1002/acn3.70499)

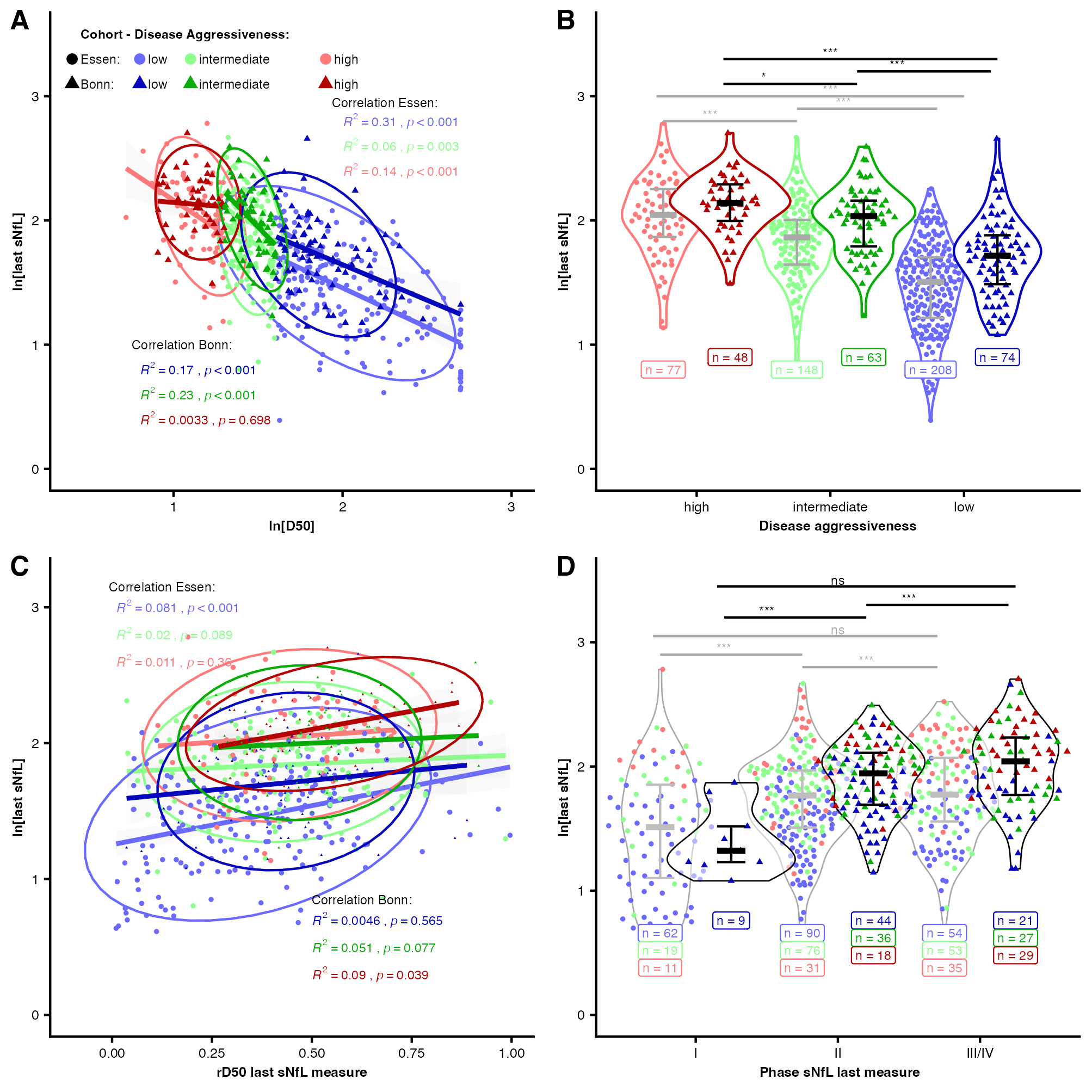

Supplement: Supplementary file 1 — Figure S1: Discovery Cohort (Essen, light Colors and circles, n = 433) and Replication Cohort (Bonn, dark colors and triangles, n = 185) with serum NfL (sNfL) at last measure. (A) Correlation of last ln[sNFL] and ln[D50] with p‐values and R2—values as well as circles as 95% Confidence‐interval and lines for linear regression for disease aggressiveness. (B) Last measured ln[sNFL] splitted into disease aggressiveness and indication of significance levels within each cohort (C) Correlation between ln[sNFL] concentrations at last measurement and relative D50 (rD50) as well as circles as 95% confidence‐interval and lines for linear regression for rD50. (D) ln[sNFL] across rD50‐defined disease phases (Phase I, Phase II, and Phase III/IV). Light colors and circles indicate the discovery cohort; dark colors and triangles indicate the independent replication cohort. Disease aggressiveness groups were defined as high (D50 < 20 months), intermediate (D50 20–40 months), and low (D50 ≥ 40 months). Statistical analyses are described in the Methods section. [file ACN3-9999-0-s001.png]

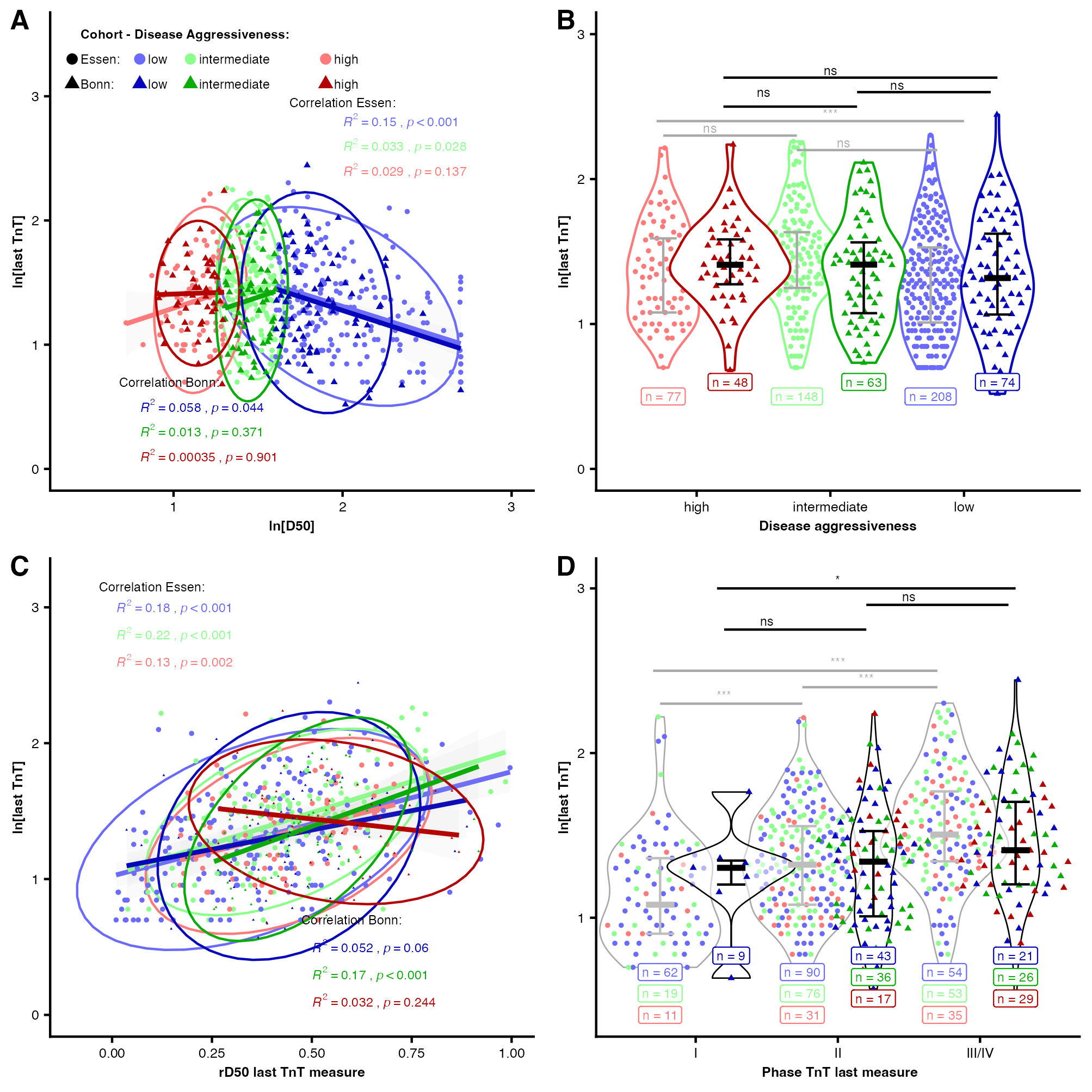

Supplement: Supplementary file 2 — Figure S2: Discovery Cohort (Essen, light Colors and circles, n = 433) and Replication Cohort (Bonn, dark Colors and triangles, n = 185) Troponin T (TnT) at last measure. (A) Correlation of last ln[TnT] and ln[D50] with p‐values and R2—values as well as circles as 95% Confidence‐interval and lines for linear regression for disease aggressiveness. (B) Last measured ln[TnT] splitted into disease aggressiveness and indication of significance levels within each cohort (C) Correlation between ln[TnT] concentrations at last measurement and relative D50 (rD50) as well as circles as 95% confidence‐interval and lines for linear regression for rD50. (D) ln[TnT] across rD50‐defined disease phases (Phase I, Phase II, and Phase III/IV). Light colors and circles indicate the discovery cohort; dark colors and triangles indicate the independent replication cohort. Disease aggressiveness groups were defined as high (D50 < 20 months), intermediate (D50 20–40 months), and low (D50 ≥ 40 months). Statistical analyses are described in the Methods section. [file ACN3-9999-0-s002.png]
