# Supplementary material for: Troponin T and Neurofilament Light Chain Levels as Complementary Biomarkers of Disease Accumulation and Aggressiveness in Amyotrophic Lateral Sclerosis
Source: Ann Clin Transl Neurol. 2026 Jul 23:10.1002/acn3.70499. Online ahead of print. doi: 10.1002/acn3.70499 (PMC13396867; doi:10.1002/acn3.70499)
Supplement: Supplementary file 3 — Table S1: ANCOVA with first sNFL and first TnT with Cofactors: Age at Onset, rD50, D50, Sex and Symptom Onset Region with effect size (Eta square and omega). [file ACN3-9999-0-s003.docx]

**Supplementary Table 1:** ANCOVA with first sNFL and first TnT with Cofactors: Age at Onset, rD50, D50, Sex and Symptom Onset Region with effect size (Eta square and omega)

| **Discovery cohort –**  **sNfL first measure** | **DF** | **F-value** | **p-value** | **Eta sq (η^2^) [95% CI]** | **Omega sq (ϖ^2^) [95% CI]** |
| --- | --- | --- | --- | --- | --- |
| **Disease aggressiveness** | 2 | 85.1775 | < 2.2e-16 *** | 0.29 \| [0.23, 1.00] | 0.28 \| [0.22, 1.00] |
| **Sex** | 1 | 8.3313 | 0.004099 ** | 0.02 \| [0.00, 1.00] | 0.02 \| [0.00, 1.00] |
| **rD50 at first sNfL measure** | 1 | 0.0043 | 0.947633 | 1.03e-05 \| [0.00, 1.00] | 0.00 \| [0.00, 1.00] |
| **Age at Onset** | 1 | 22.7905 | 2.503e-06 *** | 0.05 \| [0.02, 1.00] | 0.05 \| [0.02, 1.00] |
| **Onset Region** | 2 | 19.8525 | 5.800e-09 *** | 0.09 \| [0.05, 1.00] | 0.08 \| [0.04, 1.00] |
| **Replication cohort -sNfL first measure** | **DF** | **F-value** | **p-value** | **Eta sq (η^2^) [95% CI]** | **Omega sq (ϖ^2^) [95% CI]** |
| **Disease aggressiveness** | 2 | 31.4837 | 2.005e-12 *** | 0.26 \| [0.17, 1.00] | 0.25 \| [0.16, 1.00] |
| **Sex** | 1 | 3.9650 | 0.047995 * | 0.02 \| [0.00, 1.00] | 0.02 \| [0.00, 1.00] |
| **rD50 at first sNfL measure** | 1 | 9.3601 | 0.002563 ** | 0.05 \| [0.01, 1.00] | 0.04 \| [0.01, 1.00] |
| **Age at Onset** | 1 | 7.8399 | 0.005678 ** | 0.04 \| [0.01, 1.00] | 0.04 \| [0.00, 1.00] |
| **Onset Region** | 2 | 4.7876 | 0.009442 ** | 0.05 \| [0.01, 1.00] | 0.04 \| [0.00, 1.00] |
| **Discovery cohort– TnT first measure** | **DF** | **F-value** | **p-value** | **Eta sq (η^2^) [95% CI]** | **Omega sq (ϖ^2^) [95% CI]** |
| **Disease aggressiveness** | 2 | 2.3562 | 0.09603 | 0.01 \| [0.00, 1.00] | 6.33e-03 \| [0.00, 1.00] |
| **Sex** | 1 | 33.4355 | 1.444e-08 *** | 0.07 \| [0.04, 1.00] | 0.07 \| [0.04, 1.00] |
| **rD50 at first TnT measure** | 1 | 55.9682 | 4.384e-13 *** | 0.12 \| [0.07, 1.00] | 0.11 \| [0.07, 1.00] |
| **Age at Onset** | 1 | 6.0831 | 0.01405 * | 0.01 \| [0.00, 1.00] | 0.01 \| [0.00, 1.00] |
| **Onset Region** | 2 | 1.8960 | 0.15146 | 8.99e-03 \| [0.00, 1.00] | 4.19e-03 \| [0.00, 1.00]. |
| **Replication cohort – TnT first measure** | **DF** | **F-value** | **p-value** | **Eta sq (η^2^) [95% CI]** | **Omega sq (ϖ^2^) [95% CI]** |
| **Disease aggressiveness** | 2 | 1.5725 | 0.21056 | 0.02 \| [0.00, 1.00] | 6.46e-03 \| [0.00, 1.00] |
| **Sex** | 1 | 28.6319 | 2.833e-07 *** | 0.15 \| [0.07, 1.00] | 0.14 \| [0.07, 1.00] |
| **rD50 at first TnT measure** | 1 | 20.2298 | 1.277e-05 *** | 0.11 \| [0.04, 1.00] | 0.10 \| [0.04, 1.00] |
| **Age at Onset** | 1 | 0.3544 | 0.55241 | 2.11e-03 \| [0.00, 1.00] | 0.00 \| [0.00, 1.00] |
| **Onset Region** | 2 | 3.4794 | 0.03306 * | 0.04 \| [0.00, 1.00] | 0.03 \| [0.00, 1.00] |
| sNfL = serum Neurofilament light chain; TnT = Troponin T | | | | | |
| **Eta square** **(η^2^):** Measures the proportion of total variance in the dependent variable accounted for by an effect.  **Omega2 square** **(ϖ^2^):** A less biased estimate than (eta ^{2}), preferred for small sample sizes because it estimates the effect in the population rather than just the sample. | | | | | |
| **Interpretation**:  **Small Effect:** (0.01 $\leq$ ES < 0.06) (Variance explained: (1 % - 6 % ))  **Medium Effect:** (0.06 $\leq$ ES < 0.14) (Variance explained: (6 % - 14 %))  **Large Effect:** (ES $\geq$ 0.14) (Variance explained: (>14 % )) | | | | | |
